# Impaired neutrophil extracellular trap formation in β-thalassaemia/HbE

**DOI:** 10.1038/s41598-022-06036-7

**Published:** 2022-02-04

**Authors:** Rattanawan Thubthed, Sirikwan Siriworadetkun, Kittiphong Paiboonsukwong, Suthat Fucharoen, Kovit Pattanapanyasat, Jim Vadolas, Saovaros Svasti, Pornthip Chaichompoo

**Affiliations:** 1 Department of Pathobiology, Faculty of Science, Mahidol University, Bangkok, Thailand; 2 Thalassemia Research Center, Institute of Molecular Biosciences, Mahidol University, Nakhon Pathom, Thailand; 3 Siriraj Center of Research Excellence for Microparticle and Exosome in Diseases, Faculty of Medicine Siriraj Hospital, Mahidol University, Bangkok, Thailand; 4 Centre for Cancer Research, Hudson Institute of Medical Research, Melbourne, Australia; 5 Department of Molecular and Translational Science, Monash University, Melbourne, Australia; 6 Department of Biochemistry, Faculty of Science, Mahidol University, Bangkok, Thailand

**Keywords:** Innate immunity, Infectious diseases, Bacterial infection, Anaemia, Bacterial infection

## Abstract

Neutrophil dysfunction contributes to a high susceptibility to severe bacterial infection which is a leading cause of morbidity and mortality in β-thalassaemia/HbE, especially in splenectomised patients. This study demonstrated another abnormality of neutrophil function, namely neutrophil extracellular trap (NET) formation in splenectomised and non-splenectomised β-thalassaemia/HbE patients who had iron overload. A classification system of morphological NET formation using confocal microscopy was developed, and samples were categorized into early and late phases which were subdivided into web-like and non-web structures. At baseline, neutrophils from non-splenectomised patients (58 ± 4%) and splenectomised patients (65 ± 3%) had higher early phase NETs than those from normal subjects (33 ± 1%). As a mimic of iron overload and infection, haemin/PMA/LPS treatment led to a significant reduction of early NETs and an increase of late NETs in neutrophils from normal and non-splenectomised patients. Interestingly, neutrophils from splenectomised patients had impaired development of late NETs. This suggests that during infection bacteria might not be trapped and may spread from the site of infection resulting in higher susceptibility to severe bacterial infection in splenectomised patients.

## Introduction

β-Thalassaemia is caused by defective β-globin chain synthesis, resulting in decreased β-globin chains and an excess of unbound α-globin chains. Patients with severe anaemia require multiple blood transfusions, leading to iron overload and iron deposition in organs such as the liver and heart and resulting in eventual organ failure. Moreover, iron overload leads to cellular injury that contributes to the development of complications including immune abnormalities and severe infection^1^. Neutrophils, key effectors of innate immunity, play a role in the front-line defense against invading pathogens, especially in bacterial infection. Neutrophils are recruited to infection sites via the inflammatory response and consequently phagocytose and eliminate the pathogens. We have previously shown β-thalassaemic neutrophil dysfunction in processes such as chemotaxis and opsonophagocytosis in both patients and in a murine model^2,3^.

Neutrophil extracellular traps (NETs) are a neutrophil function that traps and kills pathogens at the infection site by releasing extracellular structures consisting of DNA and granule enzymes such as neutrophil elastase (NE). Generally, bacteria trigger NET formation via activation of NADPH oxidase (NOX2) and the subsequent stimulation of myeloperoxidase (MPO) and peptidylarginine deiminase 4 (PAD4). MPO-mediated oxidative activation of NE translocation from azurophilic granules degrades the actin cytoskeleton in the cytoplasm to block phagocytosis. PAD4 citrullinates histones enhancing chromatin decondensation. Subsequently, the nuclear membrane is damaged leading to chromatin expanding inside the cell and mixing with granular antimicrobial factors. Finally, the cell membrane breaks and chromatin fibers are released^4^. There are two types of NETs as assessed by morphology, namely web-like and non-web-like structures. However, the exact mechanisms and function of NETs are poor understood, especially the regulation of web-like and non-web-like NET formation^4^.

Iron overload and splenectomy could be important factors that affect NET formation during bacterial infection in β-thalassaemia/HbE. In this study, neutrophils isolated from β-thalassaemia/HbE patients and normal subjects were treated with haemin as a mimic of iron overload, and combination of phorbol 12-myristate 13-acetate (PMA) and lipopolysaccharides (LPS) as a mimic of infection. Confocal microscopic analysis of NE, histone H2A and nuclear DNA was performed to classify the morphological changes of NETs, which were divided into early and late phases, and further sub-divided into web-like and non-web like structures. Interestingly, neutrophils from splenectomised patients had increased early NETs but showed no progression to late NETs, while neutrophils from non-splenectomised patients had increased late web-like NETs. These finding suggest that splenectomised β-thalassaemia/HbE patients have impaired NET formation leading to the high susceptibility to infection.

## Results

### Increased NETs in neutrophils from β-thalassaemia/HbE patients is associated with iron status

**Figure 1:**
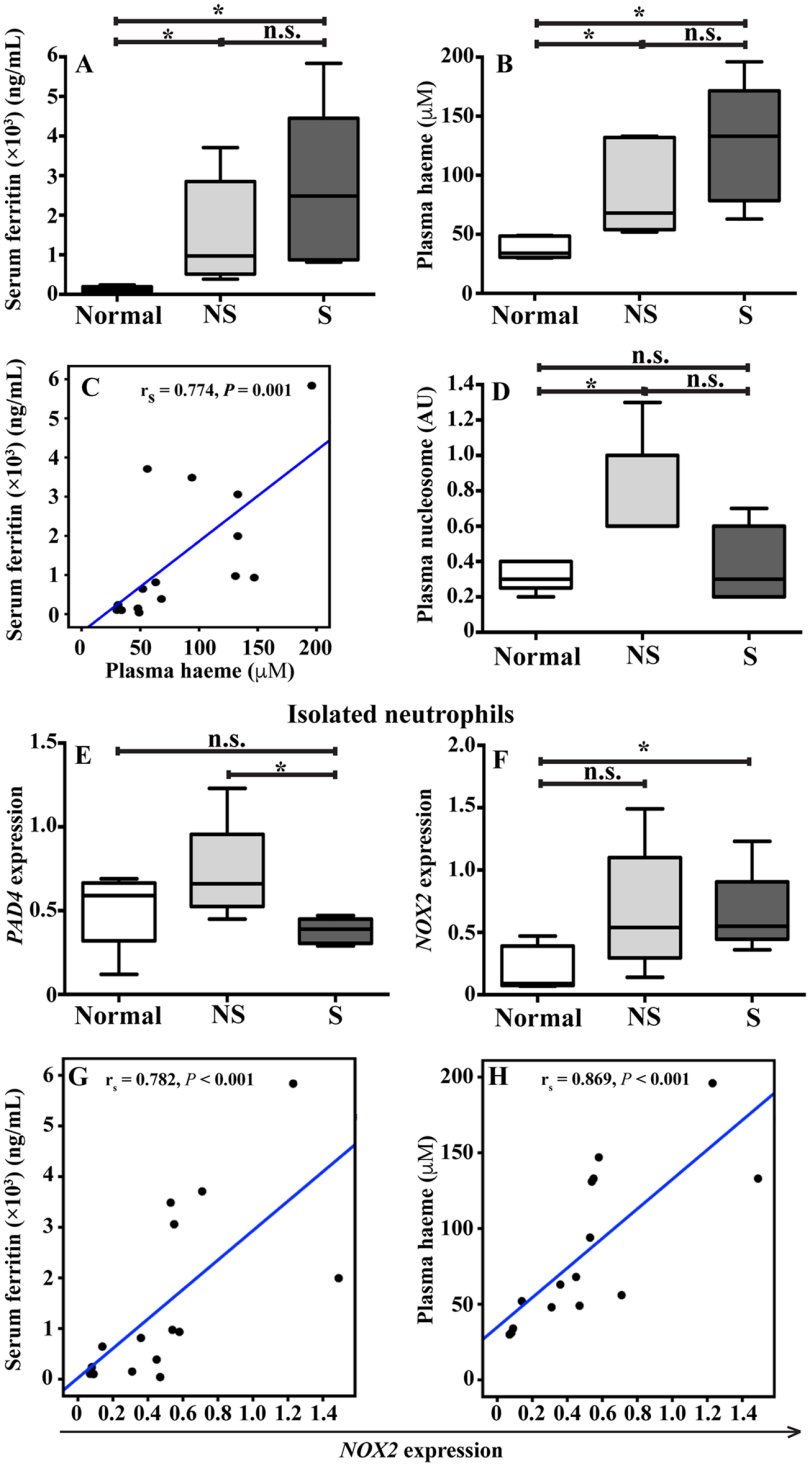
Iron overload and NET markers in β-thalassaemia/HbE. Blood samples were collected from 5 normal subjects (N), 5 non-splenectomised β-thalassaemia/HbE patients (NS) and 5 splenectomised β-thalassaemia/HbE patients (S) to determine (**A**) serum ferritin (immunoenzymatic kit, DiaMetra) and (**B**) plasma haeme (haeme assay kit, Sigma-Aldrich). (**C**) Spearman's rank correlation coefficient analysis between serum ferritin and plasma haeme. (**D**) Plasma nucleosomes were determined using the cell death detection ELISA^PLUS^ kit (Roche Diagnostics GmbH). The expression of (**E**) *PAD4* and (**F**) *NOX2* mRNA expression in isolated neutrophils from those subjects was determined using RT-qPCR. *GAPDH* was used to as normalizer to calculation of the relative quantification by 2^(−ΔCt)^ method. Spearman's rank correlation coefficient analysis between *NOX2* expression in isolated neutrophils and (**G**) serum ferritin or (**H**) plasma haeme. n.s.; no significant difference, *; significant difference at *P* < 0.05 using a non-parametric Mann–Whitney U Test.

Splenectomised β-thalassaemia/HbE patients had microcytic hypochromic anemia with leukocytosis and thrombocytosis (Supplementary Table 1). Serum ferritin and plasma haeme levels were measured as markers of iron status. Although the β-thalassaemia/HbE patients in this cohort had received iron chelation, serum ferritin and plasma haeme levels in patients were still higher than levels in normal subjects (*P* < 0.05) (Fig. 1A, B), and a significant correlation between serum ferritin levels and plasma haeme levels was observed (r_s_ = 0.774, *P* = 0.001) (Fig. 1C).

To determine the association between iron overload and NETs in β-thalassaemia/HbE patients, plasma nucleosomes and the expression of *PAD4* and *NOX2* mRNA in isolated neutrophils were examined. Non-splenectomised patients had significantly increased levels of plasma nucleosomes as compared to normal subjects (*P* < 0.05) (Fig. 1D) and significantly increased levels of *PAD4* mRNA in isolated neutrophils as compared to splenectomised patients (*P* < 0.05) (Fig. 1E). By contrast, significantly increased *NOX2* expression was seen in splenectomised patient-neutrophils as compared with normal subjects (*P* < 0.05) (Fig. 1F). Interestingly, highly significant correlations were found between *NOX2* mRNA and serum ferritin (r_s_ = 0.782, *P* < 0.001) as well as plasma haeme (r_s_ = 0.869, *P* < 0.001) (Fig. 1G, H). While serum ferritin and plasma haeme levels between splenectomised and non-splenectomised patients were not different, the expression of NET markers in splenectomised patients was different from non-splenectomised patients. Therefore, neutrophils from splenectomised and non-splenectomised patients could respond to a stimuli, such as iron overload and infection and trigger NET formation differently. Additionally, plasma nucleosome and levels of *NOX2* and *PAD4* mRNA may not truly used as an indicator of NET. Nucleosomes or histone-DNA complexes can also be used as a hallmark of cell death or necrosis. While NOX2 is critical for oxidative burst when neutrophils phagocytose pathogens. Increased *PAD4* mRNA levels may or may not mediate chromatin decondensation. Therefore, the morphological analysis of NET is needed to documented occurring of NET formation.

### Morphological classification of NET

NETs have been characterized by a number of different techniques and with different definitions, making it difficult to compare amongst reports, although a web-like morphology consisting of neutrophil chromatin protruding from the nucleus and released outside the plasma membrane has been generally accepted as a NET. However, several studies have proposed that activated neutrophils can also undergo chromatin decondensation and degranulation without the characteristic web-like NET morphology, while their releasing histone-DNA complexes and contributing to induce inflammation^4,5^. Therefore, a systematic definition of the stages of NET formation, and their morphological characteristics is needed to understand the impact of NETs on disease pathology.

**Figure 2:**
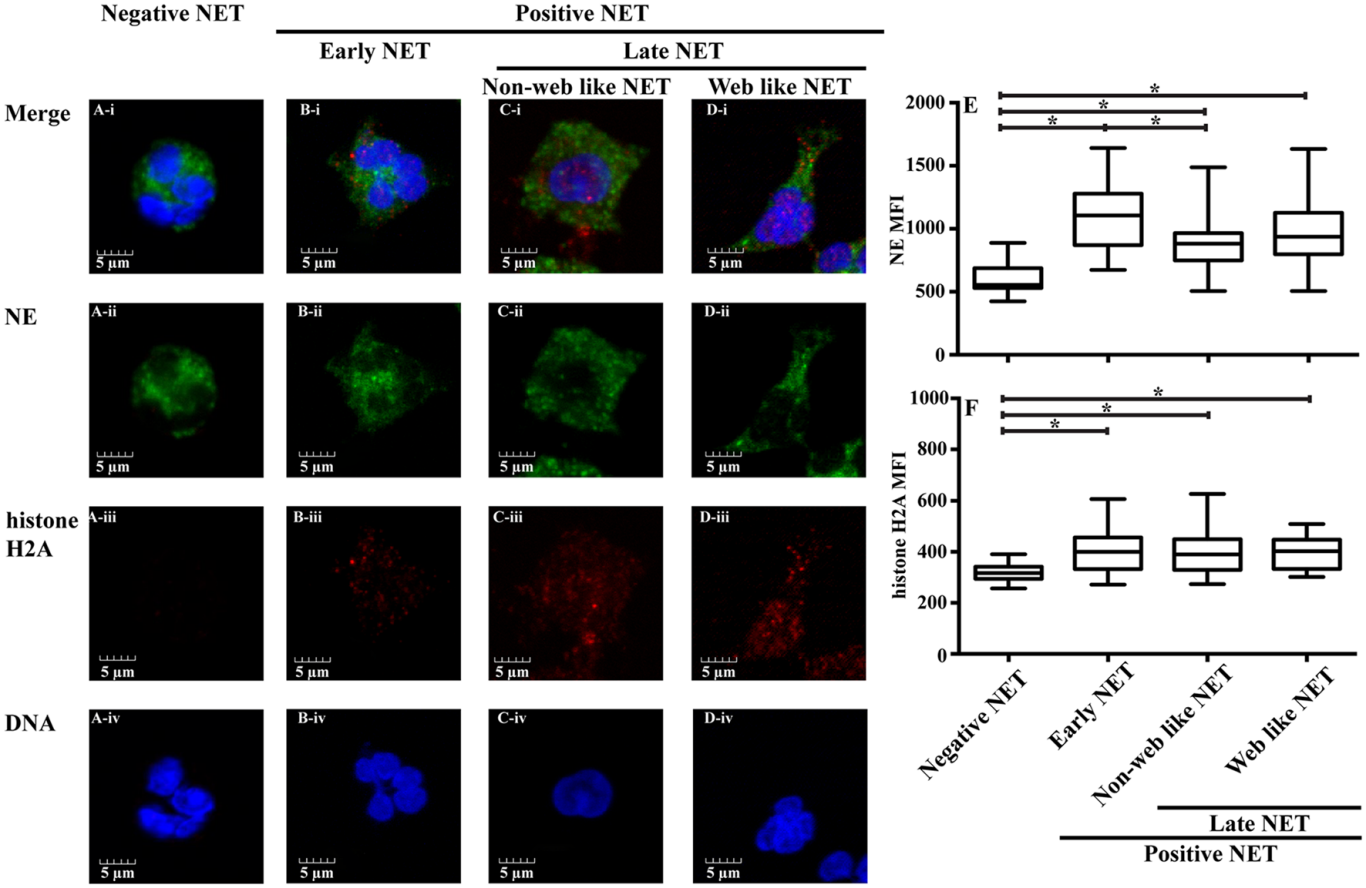
Confocal microscopic analysis of NET morphology. (**A**–**D**) Representation of confocal microscopic analysis of neutrophils from normal subject with (**A**) the absence or (**B**–**D**) the presence of combined 100 ng/mL PMA and 100 ng/mL LPS for 90 min at 37 °C, 5% CO_2_. The neutrophils were stained with fluorochrome conjugated antibodies specific to neutrophil elastase (NE, green), histone H2A (red) and nuclear DNA (blue) to analyze mean fluorescent intensity (MFI) and morphology that can identified as (**Ai–iv**) Negative NETs and (**B**–**D**) positive NETs for (**Bi–iv**) early phase, (**Ci–iv**) late phase with non-web like structure and (**Di–iv**) late phase with web-like structure. MFI of (**E)** NE and (**F**) histone H2A of different stages of NETs was analyzed from isolated neutrophils from 2 normal subjects, 2 non-splenectomised β-thalassaemia/HbE patients and 2 splenectomised β-thalassaemia/HbE patients that were incubated in the absence or the presence of combined 100 ng/mL PMA and 100 ng/mL LPS for 90 min at 37 °C, 5% CO_2_. A total of 30 neutrophils per NET stage (5 neutrophils per stage per subject) were examined from untreated and PMA/LPS-treated neutrophils for negative and positive NET, respectively. *; significant difference at *P* < 0.01 using a non-parametric Mann–Whitney U Test. i; merge, ii; anti-neutrophil elastase, iii; anti-histone H2A, iv; 4′, 6-diamidino-2-phenylindole, dihydrochloride (DAPI), LPS; lipopolysaccharides and PMA; phorbol 12-myristate 13-acetate.

In this study, a multicolor immunofluorescent assay was undertaken to determine the levels of nuclear DNA, histone H2A and NE using confocal microscopy, as previously described^6,7^ (Fig. 2). A histone-DNA complex is a major structural component of NETs, while NE is localized in granules and released during NET formation. Here, NETs were classified according to neutrophil morphological changes including number of nuclear lobulation, shape of nucleus and cell, mean fluorescent intensity (MFI) of NE and histone H2A and distance of projecting plasma membrane from nucleus to limb of plasma membrane. Negative NETs were characterized as having a segmented neutrophil with 2–5 lobes of nucleus, low level of NE MFI (mean ± S.E./median ± interquartile ranges (IQR); 630 ± 30/555 ± 158) and histone H2A MFI (317 ± 6/317 ± 48), circular in shape of cellular morphology and not present the projecting plasma membrane (Fig. 2A, E, F and Supplementary Fig. 1A). Positive NETs were divided into early and late phases. An early phase of NET development was characterized by segmented neutrophils with 2–5 lobes of nucleus, circular or irregular in shape of cellular morphology, increased NE (1,082 ± 50/1,106 ± 409) and histone H2A MFI (403 ± 16/400 ± 124) and not present projecting plasma membrane (Fig. 2B E, F and Supplementary Fig. 1B). The early phase of NET formation could be an intermediate phase in which neutrophils further develop over time to a late phase NET morphology^6^. The late phase of NET formation was further subdivided into non-web like and web like structures. Both non-web like and web like late phase of NETs were characterized by the nucleus losing its characteristic lobulation (circular shape nucleus), increased NE MFI (899 ± 40/882 ± 216 and 971 ± 48/936 ± 330, respectively) and histone H2A MFI (399 ± 15/390 ± 120 and 392 ± 12/403 ± 114, respectively) (Fig. 2C–F). The shape of neutrophils with non-web like NET had circular or irregular form and not present projecting plasma membrane or the distance of nucleus to limb of plasma membrane with length shorter than 5 μM. While web like NETs presented irregular in shape of cellular morphology and present projecting plasma membrane with the distance of nucleus to limb of plasma membrane at lengths ≥ 5 μM Supplementary Fig. 1C, D).

### Abnormal NET formation in β-thalassaemia/HbE neutrophils during infection

**Figure 3:**
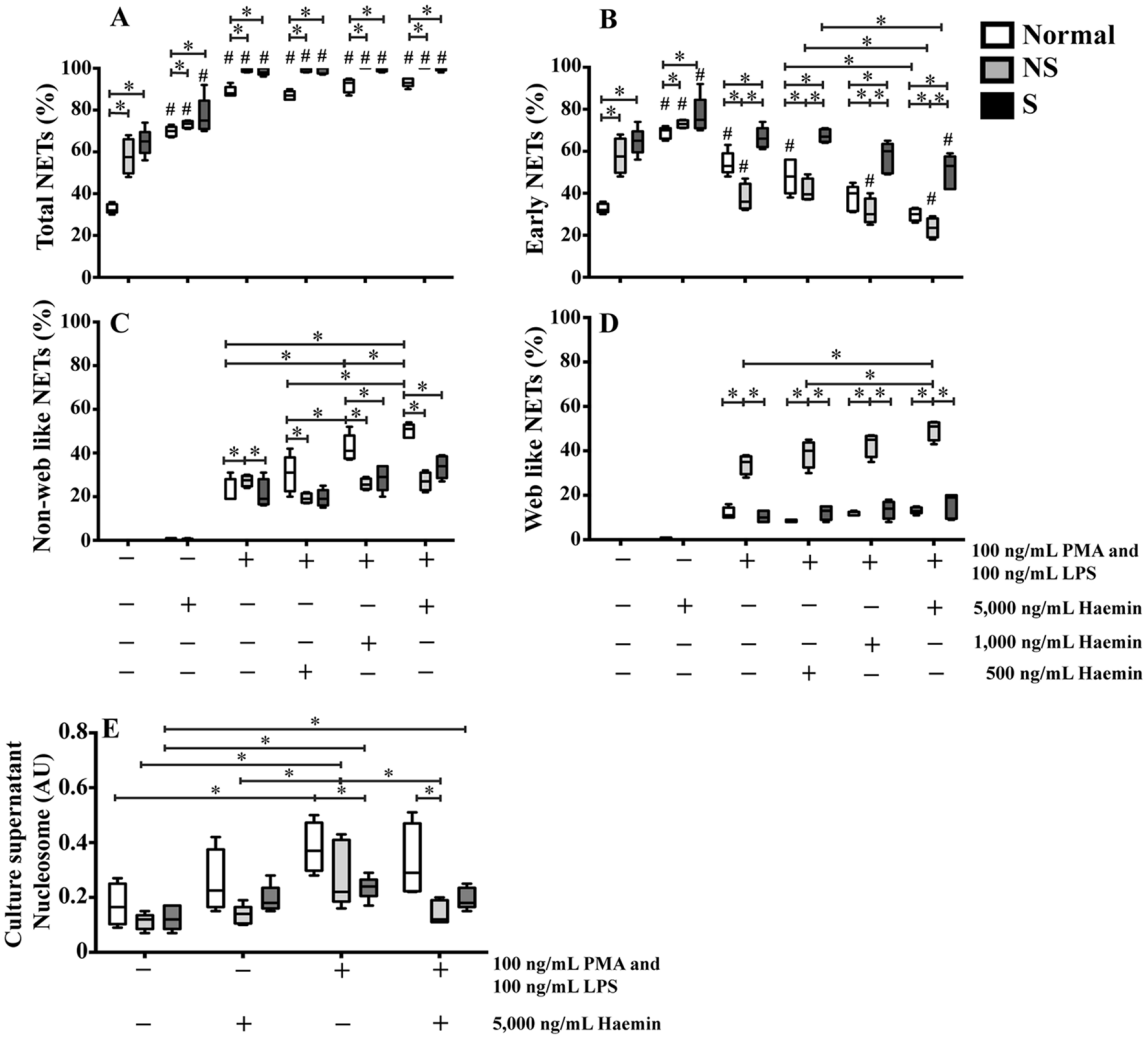
Iron overload and infection induced NET formation in β-thalassaemia/HbE patients. Isolated neutrophils from 5 normal subjects (Normal), 5 non-splenectomised β-thalassaemia/HbE patients (NS) and 5 splenectomised β-thalassaemia/HbE patients (S) were treated with either PMA and LPS or haemin or combined stimuli for 90 min at 37 °C, 5% CO_2_, then, neutrophils were fixed and stained with fluorochrome conjugated antibodies specific to neutrophil elastase (NE), histone H2A and DAPI to captured with Z-stack mode by using an Olympus confocal laser scanning microscope FV10i-DOC (Olympus Corporation, Tokyo, Japan) equipped with Olympus FluoView software. Experiments were performed as duplicated. One hundred neutrophils per condition were counted at 60 × oil lens and the percentages of (**A**) total NET formation of neutrophils, (**B**) early phase, (**C**) late phase with non-web like and (**D**) late phase with web-like structures were determined. (Supplementary Figs. 2–4) (**E**) Cell-free supernatant samples were collected after incubation to determine nucleosomes (cell death detection ELISA^PLUS^ kit, Roche Diagnostics GmbH). *Significant difference when compared between groups at *P* < 0.05. ^**#**^Significant difference when compared with unstimulated neutrophils from the individual groups at *P* < 0.05. Statistical analysis was performed by using a non-parametric Mann–Whitney U Test. DAPI; 4′, 6-diamidino-2-phenylindole, dihydrochloride.

β-Thalassaemia/HbE patients often have iron overload that could contribute to neutrophil dysfunction, therefore haemin treatment as a mimic of iron overload was investigated. Only early NETs were detected in baseline untreated neutrophils isolated from both patients and normal subjects, with significantly higher baseline NETs in splenectomised and non-splenectomised patients (mean ± SE/median ± IQR% of neutrophils; 65 ± 3/65 ± 10% and 58 ± 4/58 ± 16%, respectively) compared to normal subjects (33 ± 1/32 ± 4%) (*P* < 0.05) (Fig. 3A–D and Supplementary Fig. 1–3). There was a two-fold increase in NETs in haemin-treated neutrophils from normal subjects (70 ± 1/70 ± 5%) (*P* < 0.05). In addition, although a smaller effect as compare to normal neutrophils, significantly increased NETs were observed in haemin-treated neutrophils from splenectomised and non-splenectomised patients (77 ± 4/75 ± 14% and 73 ± 1/74 ± 3%, respectively) when compared to untreated neutrophils (*P* < 0.05). Interestingly, only early NETs were found in haemin-treated neutrophils of all groups (Fig. 3A–D).

To study NET formation during infection, the pattern of NETs after PMA/LPS treatment as a mimic of infection was determined. Significantly increased total NETs in PMA/LPS-treated neutrophils from both patients and normal subjects (98 ± 1/98 ± 3% in splenectomised patients, 99 ± 1/99 ± 1.5% in non-splenectomised patients and 89 ± 1/88 ± 4% in normal subjects) were found as compared to untreated neutrophils (*P* < 0.05) (Fig. 3A). The major type of NETs in PMA/LPS-treated neutrophils from splenectomised patients was early phase NETs (66 ± 2/66 ± 9%), which was significantly higher as compared to non-splenectomised patients (38 ± 3/36 ± 12%) and normal subjects (54 ± 2/53 ± 9%) (*P* < 0.05) (Fig. 3B). In contrast, neutrophils from non-splenectomised patients had significantly higher late phase NETs (61 ± 3/63 ± 12%) with web-like NETs as the majority (34 ± 2/35 ± 8%) (Fig. 3D).

The effect of iron overload on neutrophil responsiveness to infection in β-thalassaemia/HbE was then examined by co-incubating neutrophils with haemin and PMA/LPS. Early NETs were significant decreased and non-web-like late NETs were significantly increased in neutrophils from normal subjects and splenectomised patients treated with haemin/PMA/LPS in a haemin dose-dependent manner. Nevertheless, splenectomised patients' neutrophils had a reduced degree of early NETs, and increased non-web-like late NETs as compared to normal subjects, while the web-like late NETs were not significantly different between PMA/LPS-treated and haemin/PMA/LPS-treated neutrophils of both groups (Fig. 3B–D). By contrast, neutrophils from non-splenectomised patients had significantly more web-like late NETs than non-web-like NETs after haemin/PMA/LPS-treatment. At the highest doses of haemin/PMA/LPS treatment, early NETs in neutrophils from splenectomised patients (50 ± 4/53 ± 16%) were significant higher than in non-splenectomised patient neutrophils (24 ± 2/24 ± 9%) and in neutrophils from normal subjects (30 ± 1/30 ± 6%). In contrast, the majority of NETs in neutrophils from normal and non-splenectomised patients were non-web-like late NETs (50 ± 1/51 ± 6%) and web-like late NETs (50 ± 2/51 ± 8%), respectively (Fig. 3B–D). The decreased late NETs in neutrophils from splenectomised patients could imply neutrophil dysfunction in response to infection.

When NET or necrosis occurred, the nucleosomes or the histone-DNA complexes could be released out of cells. It is still unclear that levels of nucleosome can also be used as a marker of NET. In this regard, the supernatant nucleosomes in untreated and either haemin or PMA/LPS or combined haemin/PMA/LPS-treated neutrophils from patients and normal subjects were examined. Significant increased supernatant nucleosomes in PMA/LPS-treated neutrophils compared to untreated-neutrophils from the individual groups were found, however, there was no correlation between levels of the supernatant nucleosomes and the percentages and the morphology of NET in this study (Fig. 3E).

### Clinical parameters related to NET formation in β-thalassaemia/HbE

Multiple regression analysis was undertaken to determine whether there were clinical parameters related to NET formation in neutrophils from β-thalassaemia/HbE patients. Splenectomy and serum ferritin or splenectomy and plasma haeme play roles in NET formation explaining about 80% of the variation (Table 1). In addition, neutrophil *NOX2* expression also contributed to NET formation, with *NOX2* expression combined with splenectomy and plasma haeme explaining 92.6% of variation of NET formation (*R* = 0.962, *P* < 0.001).

**Table 1:** A multivariate analysis of NET formation and clinical parameters.

Dependent (Y)	Independent (X)	R	*P*-value	R^2^ (%)	Regression equation ${\hat{\text{y}}} = {\text{b}}_{0} + {\text{b}}_{1} {\text{x}}$
NET formation^a^	Splenectomy Serum ferritin	0.894	< 0.001	80.0	Y = 19.584 + (7.271 × 10^−5^)SF + 15.802Sp
	Splenectomy Plasma haeme	0.896	< 0.001	80.3	Y = 19.533 + 14.777Sp + 0.025Haeme
	Splenectomy *NOX2* expression^a^	0.952	< 0.001	90.5	Y = 18.178 + 21.091Sp-120.726NOX
	Splenectomy Plasma haeme *NOX2* expression^a^	0.962	< 0.001	92.6	Y = 18.431 + 18.475Sp-129.774NOX + 0.068Haeme

NOX; *NOX2* expression, SF; serum ferritin, and Sp; splenectomy. The splenectomy was scoring by 1 for normal subjects (non-splenectomised), 2 for non-splenectomised β-thalassaemia/HbE patients and 3 for splenectomised β-thalassaemia/HbE patients.

^a^NET formation and *NOX2* expression were analyzed from spontaneously untreated neutrophils in culture media at 90 min 37ºC, 5%CO2.

## Discussion

β-Thalassaemia/HbE patients are highly susceptible to severe infection, contributing to morbidity and mortality. Severe anaemia, iron overload and splenectomy are important factors that can induce many complications, including immune abnormalities, in patients^3,8^. Neutrophil dysfunction in both murine models and patients with β-thalassaemia has been observed, including reduction of neutrophil chemotaxis and opsonophagocytosis due to the reduced expression of extracellular molecules involved in phagocytosis, inflammation and migration^2,3,9^. NETs are a well-known neutrophil response to infection that traps microorganism at the site of infection limiting microorganism spread. This study is the first report showing that neutrophils from β-thalassaemia/HbE patients have impaired NET formation associated with iron overload and splenectomy.

Several techniques have been used for the analysis of NETs, such as electron microscopy and immunofluorescent staining. Electron microscopy is used to provide a high-resolutional morphological analysis of the nuclear lobular complexes in neutrophils^10^. However, this technique needs special skill, an expensive instrument and as well as a classification guideline for defining non-web-like NETs. Flow cytometric analysis of immunofluorescent double staining of the expression of H3citrulline and MPO has been employed, but cell clumping in the late phase of NET formation limits the usefulness of this analytical technique^11^. Confocal microscopic analysis of immunofluorescent staining for NET markers such as NE, MPO, H1/H2A/H3citrulline histone and nuclear DNA with neutrophil morphology has the advantages of measuring the percentage of NETs and quantifying the area of NET formation^6^. However, the limitation of this technique is that it cannot be applied to high throughput screening. This study is the first report on the classification of NETs into the early and late phases of NET formation, subdivided into web-like and non-web-like NETs. This classification may help in understanding the process of NET formation, in understanding whether non-web-like NETs may be an intermediate stage before development of web-like NETs.

The defective progression to late NETs of neutrophils from splenectomised β-thalassaemia/HbE patients could be one of the factors that cause an increased susceptibility to infection as microbes might more readily spread from the site of infection. A previous study has shown that thalassaemic patients who had undergone splenectomy > 10 years showed a significantly increased risk of bacterial infection with an odds ratio of 4.0 (95%CI 1.1–14; *P* = 0.02) when compared to non-splenectomised patients^12^. Although neutrophils from non-splenectomised patients and normal subjects presented late NETs after induction with haemin/PMA/LPS, the form of NETs were different. However, the mechanisms and function of web-like and non-web-like NETs are still unclear. The formation of NETs can be triggered by several factors such as microorganisms and endogenous stimuli such as immune complexes, calcium, cholesterol crystals and haeme^4^. Therefore, neutrophils from non-splenectomised patients had an increased responsiveness to haemin/PMA/LPS treatment to form the web-like NETs as compared to neutrophils from normal subjects, which could result from neutrophils primed by iron overload and chronic inflammation. Moreover, the baseline of *PAD4* expression in neutrophils from non-splenectomised patients was higher than in neutrophils from splenectomised patients. However, neutrophil pathology and immune-related complications in β-thalassaemia/HbE disease need further study.

Nucleosomes have also been used as a marker for NETs^13^. Increased nucleosome levels were increased in PMA/LPS-treated neutrophils. However, nucleosome levels were not correlated with the morphological changes of NETs. Nucleosomes can be released from cells by several mechanism such as necrosis, apoptosis and by NETs^14^. These results suggest that the levels of nucleosomes are not specific markers for NETs. The morphological changes of neutrophils could be more useful in determination of NETs, while supernatant nucleosomes might be used as an optional detection methodology.

Iron overload is one of the factors that is associated with neutrophil dysfunction. Haemin treatment did not activate late NET formation, but haemin could prime neutrophils to undergo a rapid response to further stimuli such as stress, inflammation or infection. Inflammatory cytokines and reactive oxygen species (ROS) have been reported to induced NET formation^4,15^. It is noteworthy that β-thalassaemia/HbE patient neutrophils have increased ROS^16^ and patient have increased inflammatory cytokines such as M-CSF, TNF-α, IFN-γ and IL-1β in circulation^17–19^. TNF-α treated normal neutrophils in plasma from sickle cell disease patients contain increased haeme levels, and increased web-like NETs as compared to neutrophils in plasma from allologous normal subjects^13^. Additionally, deferasirox, an iron-chelating agent reduced ROS production and NET formation in PMA-treated neutrophils from normal subjects^20^.

In conclusion, the classification of stages of NETs using an immunofluorescent assay as developed here could be useful for studies of neutrophil function. Neutrophil dysfunction in β-thalassaemia/HbE patients including a reduction in chemotaxis and opsonophagocytosis has been observed. In this study, another abnormality of the neutrophil response to infection, namely NET formation, was elucidated. Neutrophils from patients had a response to haeme and infection that was different to neutrophils from normal controls, which could occur as a result of cellular stress and spleen function. Impaired development of the late phase of NET formation in splenectomised patients could be associated with severe infection. However, the mechanism of NET formation contributing to neutrophil dysfunction in β-thalassaemia/HbE needs further study.

## Materials and methods

### Patients and blood samples

Ten β-thalassaemia/HbE patients (five non-splenectomised and five splenectomised) and five normal subjects with ages ranging from 19 to 46 years were recruited. The study protocol was approved by the Mahidol University Institutional Review Board (approval number COA.No.2017/043.1403 and 2021/069.2503). Written informed consent was obtained from all individual participants included in the study. All subjects had no evidence of current infection. Inclusion and exclusion criteria of patients in this cohort study are described in Supplementary Methods. All methods were performed in accordance with the relevant guidelines and regulations.

Blood samples were collected in citrate–phosphate-dextrose-adenine solution anticoagulant tubes at room temperature and were processed within 2–3 h. Measurement of serum ferritin (immunoenzymatic kits, DiaMetra, Segrate, Italy), plasma haeme (haeme assay kit, Sigma-Aldrich, St Louis, MO, USA) and plasma/supernatant nucleosomes (cell death detection ELISA^PLUS^ kit, Roche Diagnostics GmbH, Mannheim, Germany) were undertaken as detailed in the manufacturer's instructions. Haematological parameters of the samples examined in this study are shown in Supplementary Table 1.

### Neutrophil isolation and treatment

Peripheral blood neutrophils were isolated as described in a previous study^2^ (Supplementary Methods). Isolated neutrophils were activated with 100 ng/mL PMA (Sigma-Aldrich), 100 ng/mL LPS (Sigma-Aldrich) and different doses of haemin (500–5000 ng/mL) (Sigma-Aldrich) for 90 min at 37 °C, 5%CO_2_.

### Analysis of neutrophil extracellular trap

Isolated neutrophils were used to determine the expression of *NOX2*, *PAD4* and *GAPDH* mRNA using RT-qPCR. Relative quantification of *NOX2* and *PAD4* was normalised with *GAPDH* and calculated by following the 2^(−∆Ct)^ method. Morphology and percentage of NETs of isolated neutrophils with or without treatment were determined by the expression of NE, histone H2A and nuclear DNA using a confocal microscope (Supplementary Methods).

### Redundant publication

No substantial overlap with previous papers.

## Supplementary Information

Supplementary Information.

## Abbreviations

NET: Neutrophil extracellular trap
NOX2: NADPH oxidase
PAD4: Peptidylarginine deiminase 4
