# Supplementary material for: Impaired neutrophil extracellular trap formation in β-thalassaemia/HbE
Source: Sci Rep. 2022 Feb 4;12:1967. doi: 10.1038/s41598-022-06036-7 (PMC8816948; doi:10.1038/s41598-022-06036-7)
Supplement: Supplementary file 1 — Supplementary Information. [file 41598_2022_6036_MOESM1_ESM.pdf]

Supplementary information

Impaired neutrophil extracellular trap formation in β-thalassaemia/HbE

Rattanawan Thubthed[1], Sirikwan Siriworadetkun[1,2], Kittiphong Paiboonsukwong[2], Suthat Fucharoen[2], Kovit Pattanapanyasat[3], Jim Vadolas[4,5], Saovaros Svasti[2,6], Pornthip Chaichompoo[1,*]

[1]Department of Pathobiology, Faculty of Science, Mahidol University, Bangkok, Thailand; [2]Thalassemia Research Center, Institute of Molecular Biosciences, Mahidol University, Nakhon Pathom, Thailand; [3]Siriraj Center of Research Excellence for Microparticle and Exosome in Diseases, Faculty of Medicine Siriraj Hospital, Mahidol University, Bangkok, Thailand; [4]Centre for Cancer Research, Hudson Institute of Medical Research, Melbourne, Australia; [5]Department of Molecular and Translational Science, Monash University, Melbourne, Australia; and [6]Department of Biochemistry, Faculty of Science, Mahidol University, Bangkok, Thailand.

*Correspondence to:

Pornthip Chaichompoo, PhD

Department of Pathobiology, Faculty of Science, Mahidol University, 272 RamaVI Rd., Ratchathewi, Bangkok 10400 Thailand.

Tel.: +66 2 201 5577, Fax: +66 2 354 7158 E-mail: pornthip.chh@mahidol.ac.th

Supplementary Methods: 1

Number of supplementary table: 1

Number of supplementary figure: 4

Supplementary Methods

Patients

Patients who were under treatment with prednisolone or antibiotics had excluded from this study, and none of the patients were hospitalised or transfused within the proceeding 4 weeks. All patients received daily folic acid for 5 mg/day, deferiprone (GPO-L-ONE®, the Government Pharmaceutical Organization, Thailand) for 50-100 mg/kg/day and hydroxyurea (Hydrea, Corden Pharma Latina S.p.A., Italy) for 5-10 mg/kg/day.

Neutrophil isolation and treatment

Peripheral blood neutrophils were isolated by gradient centrifugation using Lymphoprep™ solution (Fresenius/Axis-Shield PoC AS, Oslo, Norway) and 6% dextran solution (Sigma-Aldrich) as described in previous study[1]. Isolated neutrophils (2×10^5 cells) in RPMI1640 media (Gibco, Paisley, UK) were activated with 100 ng/mL phorbol 12-myristate 13-acetate (PMA) (Sigma-Aldrich), 100 ng/mL lipopolysaccharides (LPS) (Sigma-Aldrich) and different doses of haemin (500 to 5,000 ng/mL) (Sigma-Aldrich) in 24-well-plates for 90 min at 37°C, 5%CO_2. Untreated neutrophils were used as baseline control. After incubation, the percentages of NETs, expression of *NOX2* and *PAD4* mRNA, and cell-free supernatant nucleosomes were examined.

NOX2 and *PAD4* expression

Total RNA was extracted from isolated neutrophils by using TRIzol™ agent (Invitrogen, CA, USA). cDNA was reverse transcribed by using MultiScribe™ high-capacity cDNA reverse transcription kit (Invitrogen). The iTaq™ universal SYBR® Green supermix (Bio-Rad, CA, USA) was used to perform RT-qPCR analysis for *NOX2*, *PAD4* and *GAPDH* with the ABI 7500 system (Applied Biosystems, CA, USA). Relative quantification of *NOX2* and *PAD4* was normalised with *GAPDH* and calculated by following $2^{(-\Delta Ct)}$ method. The sequences of primers were as follows; *NOX2* forward: 5'-ACTTCTTGGGTCAGCACTG-3', reverse: 5'-ATTCCTGTCCAGTTGTCTTCG-3'. *PAD4* forward: 5'- TTGACAGCGAAGA CCTGCA-3', reverse: 5'-ACCCAAGACTACGCTGCACT-

3'. *GAPDH* forward: 5'- TGCACCACCAACTGCTTAGC-3', reverse: 5'-GGCATGGACTGTGGTCA TGAG-3'.

Analysis of neutrophil extracellular trap formation

Indirect intracellular immunofluorescence of isolated neutrophils from β-thalassaemia/HbE patients and normal subjects was performed to analysis the morphology and the percentages of NETs. Briefly, attached neutrophils on 13 mm glass coverslips after PMA, LPS and haemin treatments were fixed with 4% paraformaldehyde solution (Sigma-Aldrich). After permeated and blocked neutrophils were incubated with a mouse anti-neutrophil elastase monoclonal antibody (Thermo Scientific, IL, USA) and a rabbit anti-histone H2A polyclonal antibody (Thermo Scientific) and subsequently a FITC-conjugated goat anti-mouse IgG polyclonal antibody (Thermo Scientific), a Cy5-conjugated goat anti-rabbit IgG polyclonal antibody (Thermo Scientific) and 4′, 6-diamidino-2-phenylindole, dihydrochloride (DAPI) (Molecular Probes, OR, USA). Fluorescence confocal microscope images were captured with Z-stack mode by using an Olympus confocal laser scanning microscope FV10i-DOC (Olympus Corporation, Tokyo, Japan) equipped with Olympus FluoView software. Illustration was captured and count 100 neutrophils per specimen at 60× oil lens. Experiments were duplicated.

Statistical analysis

Data were analysed using SPSS version 18.0 (IBM Collaboration, Chicago, IL, USA) and GraphPad PRISM 6.0 (GraphPad Software, San Diego, CA, USA) software. Comparisons between parameters were evaluated with a non-parametric Mann-Whitney U Test. The correlation coefficient between parameters were calculated with Spearman's Rho (r_s). The threshold for statistical significance for all comparisons was $P<0.05$.

Reference

1 Siwaponanan, P. *et al.* Reduced PU.1 expression underlies aberrant neutrophil maturation and function in beta-thalassemia mice and patients. *Blood* **129**, 3087-3099 (2017).

Supplementary Table 1. Haematological parameters

Description	Normal subjects	β-Thalassaemia/HbE patients		Reference range
		Non-splenectomy	Splenectomy	
Number (Male: Female)	5 (4: 1)	5 (2: 3)	5 (3: 2)	
Age (years) (range)	29±7 (22-37)	39±6 (31-46)	31±9 (19-43)	
Red blood cell count (×10^6/μL)	5.1 ± 0.6	3.5 ± 0.6[a]	3.1 ± 0.5[a]	4.2-5.4
Haemoglobin (g/dL)	14.7 ± 1.5	7.8 ± 1.3[a]	6.6 ± 0.9[a]	12-18
Haematocrit (%)	44.7 ± 4.5	23.7 ± 3.4[a]	21.2 ± 1.9[a]	37-52
MCV (fL)	87.9 ± 4.3	67.3 ± 2.0[a]	69.0 ± 5.0[a]	80-99
MCH (pg)	28.9 ± 1.4	22.2 ± 2.5[a]	21.5 ± 1.7[a]	27-31
MCHC (g/dL)	32.9 ± 0.3	32.9 ± 2.0	30.9 ± 2.4	31-35
Red cell distribution width (%)	12.4 ± 0.7	28.4 ± 3.6[a]	30.1 ± 2.3[a]	11.5-14.5
NRBCs (cells/100WBCs)	0 ± 0	3.8 ± 2.3[a]	144.8 ± 17.7[a,b]	None
WBC count (×10^3/μL)	6.3 ± 1.5	6.1 ± 1.4	13.7 ± 5.2[a,b]	4-11
Absolute neutrophils (×10^3/μL)	3.6 ± 1.2	3.5 ± 0.8	5.2 ± 2.2	1.5-8.0
WBC differential count				
Band form (%)	0 ± 0	0 ± 0	0 ± 0	0-1
Neutrophils (%)	57 ± 7	54 ± 7	37 ± 8	40-74
Eosinophils (%)	2 ± 1	2 ± 2	5 ± 5	0-7
Basophils (%)	0 ± 0	0 ± 0	0 ± 0	0-1.5
Lymphocytes (%)	35 ± 7	38 ± 8	50 ± 11	20-48
Monocytes (%)	4 ± 1	4 ± 3	8 ± 2	2-10
Platelet count (×10^3/μL)	229 ± 53	280 ± 158	694 ± 72[a,b]	150-450
Serum ferritin (ng/mL)	127 ± 73	1,541 ± 1,357[a]	2,826 ± 2,073[a]	12-300
Plasma haeme (μM)	38 ± 9	88 ± 41 [a]	127 ± 51 [a]	38-42
Plasma nucleosome (AU)	0.3 ± 0.1	0.8 ± 0.3 [a]	0.4 ± 0.2	0-0.3

AU; absorbent unit, MCH; mean corpuscular haemoglobin, MCHC; mean corpuscular haemoglobin concentration, MCV; mean corpuscular volume, NRBCs; nucleated red blood cells and WBC; white blood cells. Data presents Mean±SD. [a]Significant different when compared to normal subjects at $P < 0.05$. [b]Significant different when compared to non-splenectomised β-thalassaemia/HbE patients at $P < 0.05$.

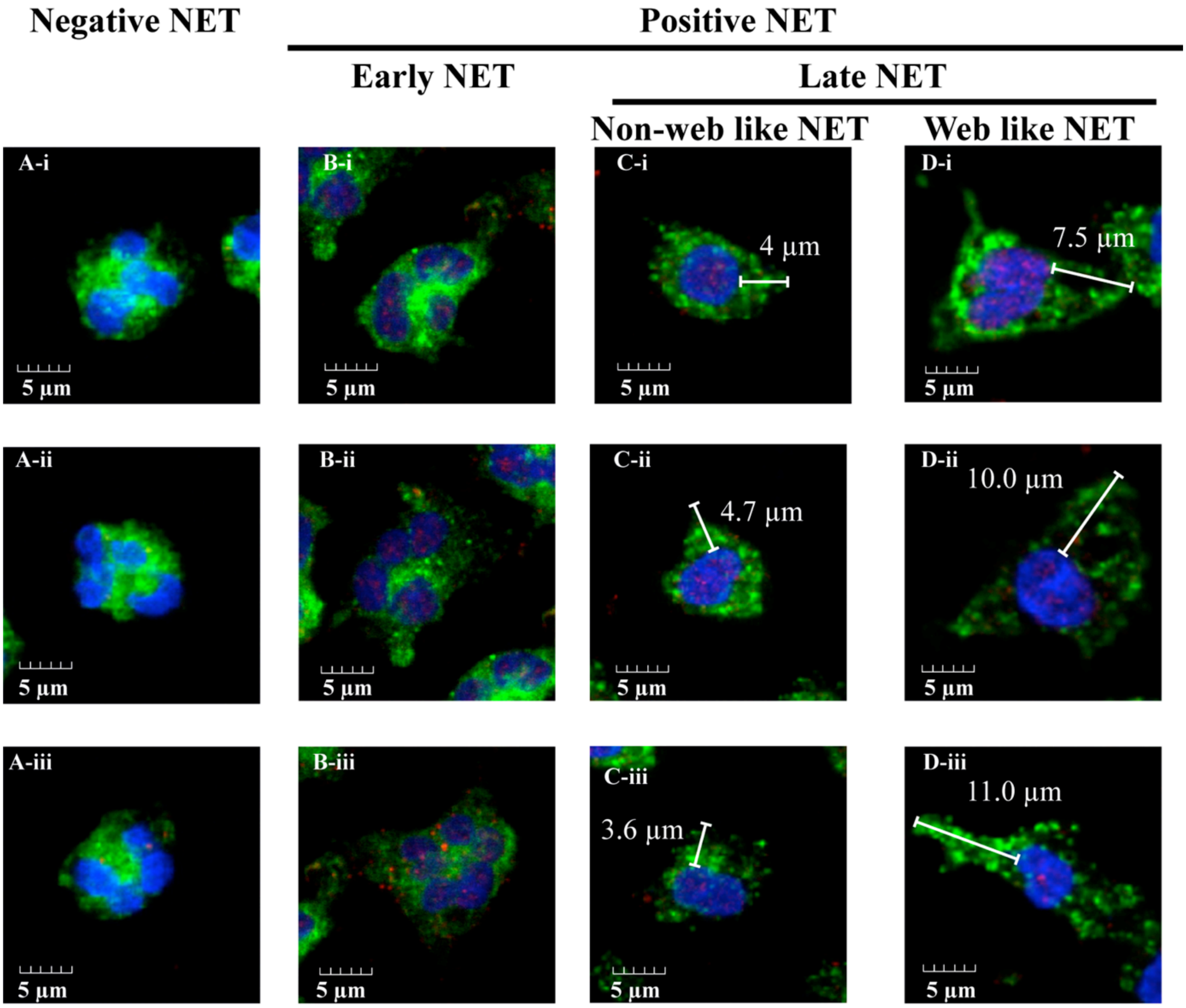

Supplementary Figure 1. Morphological classification of NETs. Isolated neutrophils from normal subjects were incubated in either RPMI1640 media as baseline control or media containing 100 ng/mL PMA and 100 ng/mL LPS as activated NET formation for 90 min at 37ºC, 5% CO_2, then, neutrophils were fixed and performed the indirect intracellular immunofluorescent assay using monoclonal antibody specific to neutrophil elastase (NE, green), histone H2A (red) and nuclear DNA (DAPI) (blue). Illustration was captured using confocal microscope. The criteria for NET classification was considered the lobulation, mean fluorescent intensity (MFI) of NE and histone H2A and projecting plasma membrane. (A) Neutrophils with negative NET had 2-5 lobes of nucleus, circular in shape, low level of NE and histone H2A MFI and not present projecting plasma membrane. (B) Neutrophils with early phase of NET had 2-5 lobes of nucleus, circular or irregular in shape, increased NE and histone H2A MFI and not present projecting plasma membrane. (C) Neutrophils with non-web like late phase of NET had circular in shape of nucleus, no lobulation of nucleus, circular or irregular in shape of cellular morphology, increased NE and histone H2A MFI and not present projecting plasma membrane or the distance of nucleus to limb of plasma membrane with length shorter than 5 µM. (D) Neutrophils with web like late phase of NET had circular in shape of nucleus, no lobulation of nucleus, irregular in shape of cellular morphology, increased NE and histone H2A MFI and present projecting plasma membrane with the distance of nucleus to limb of plasma membrane at length ≥ 5 µM. i-iii; there were 3 different neutrophils, Ai-Aiii; untreated neutrophils, B-D; PMA/LPS-treated neutrophils. DAPI; 4′, 6-diamidino-2-phenylindole, dihydrochloride, LPS; lipopolysaccharides and PMA; phorbol 12-myristate 13-acetate.

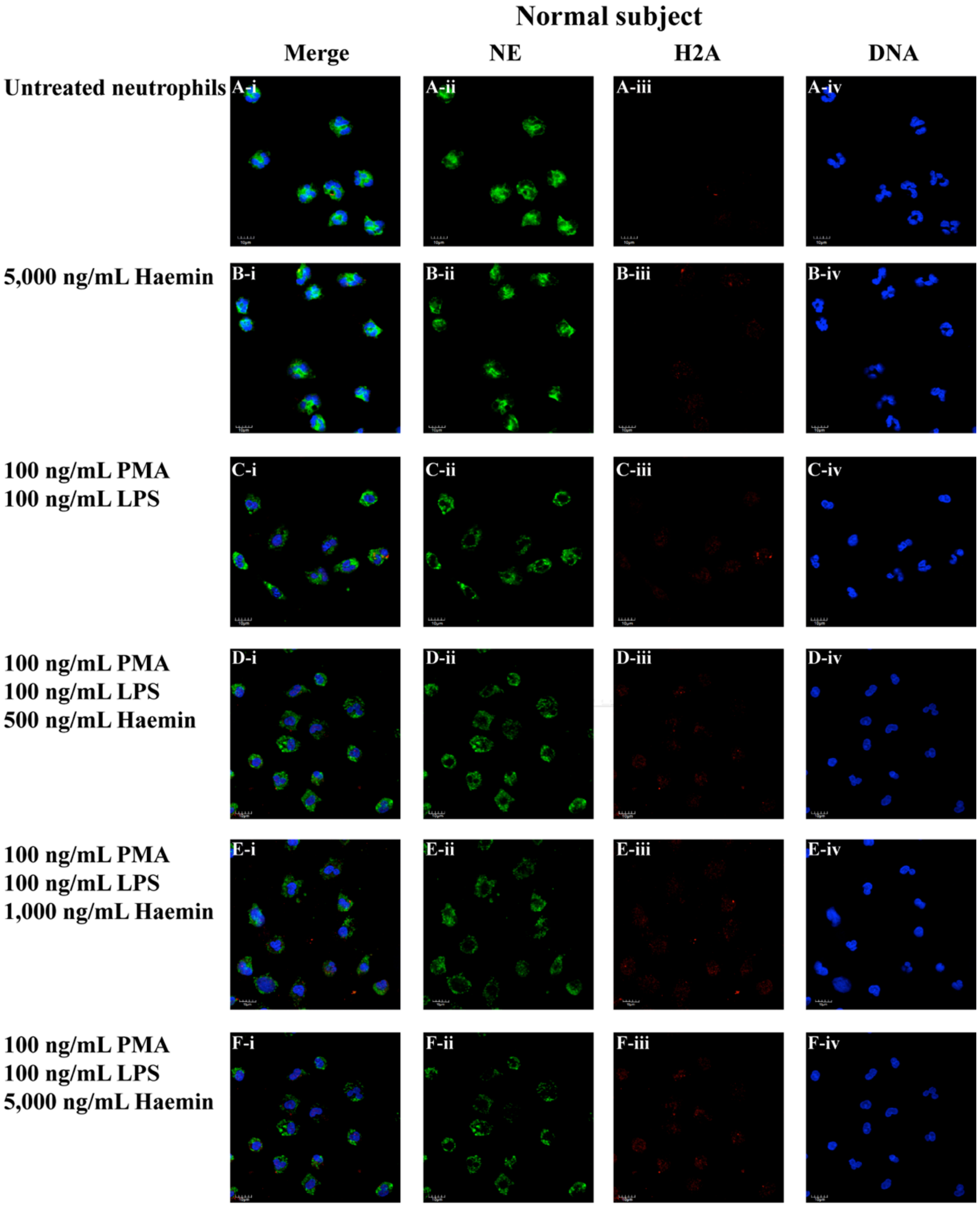

Supplementary Figure 2. NET formation in normal subjects. Isolated neutrophils from peripheral blood samples were incubated with absence or present of combined haemin and PMA/LPS for 90 min at 37°C, 5% CO_2. (Ai-iv) Untreated neutrophils, (Bi-iv) haemin-treated neutrophils and (C-F) combined PMA/LPS and different doses of haemin treated neutrophils were stained with fluorochrome conjugated antibodies specific to neutrophil elastase (NE, green), histone H2A (red) and nuclear DNA (blue) to analyze fluorescent intensity and morphology. i; merge, ii; anti-neutrophil elastase, iii; anti-histone H2A, iv; 4′, 6-diamidino-2-phenylindole, dihydrochloride (DAPI), LPS; lipopolysaccharides and PMA; phorbol 12-myristate 13-acetate.

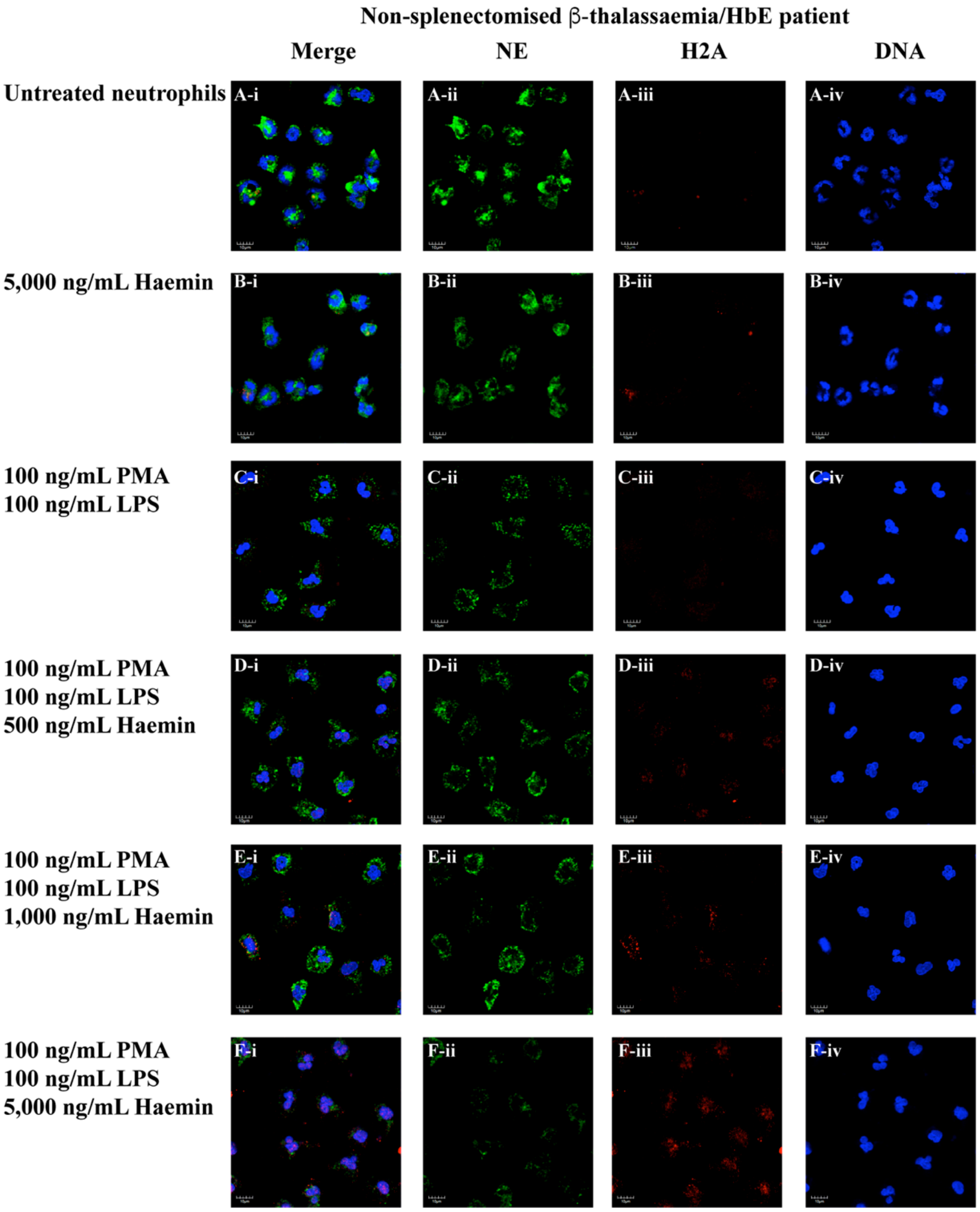

Supplementary Figure 3. NET formation in non-splenectomised β-thalassaemia/HbE patients. Isolated neutrophils from peripheral blood samples were incubated with absence or present of combined haemin and PMA/LPS for 90 min at 37°C, 5% CO_2. (Ai-iv) Untreated neutrophils, (Bi-iv) haemin-treated neutrophils and (C-F) combined PMA/LPS and different doses of haemin treated neutrophils were stained with fluorochrome conjugated antibodies specific to neutrophil elastase (NE, green), histone H2A (red) and nuclear DNA (blue) to analyze fluorescent intensity and morphology. i; merge, ii; anti-neutrophil elastase, iii; anti-histone H2A, iv; 4′, 6-diamidino-2-phenylindole, dihydrochloride (DAPI), LPS; lipopolysaccharides and PMA; phorbol 12-myristate 13-acetate.

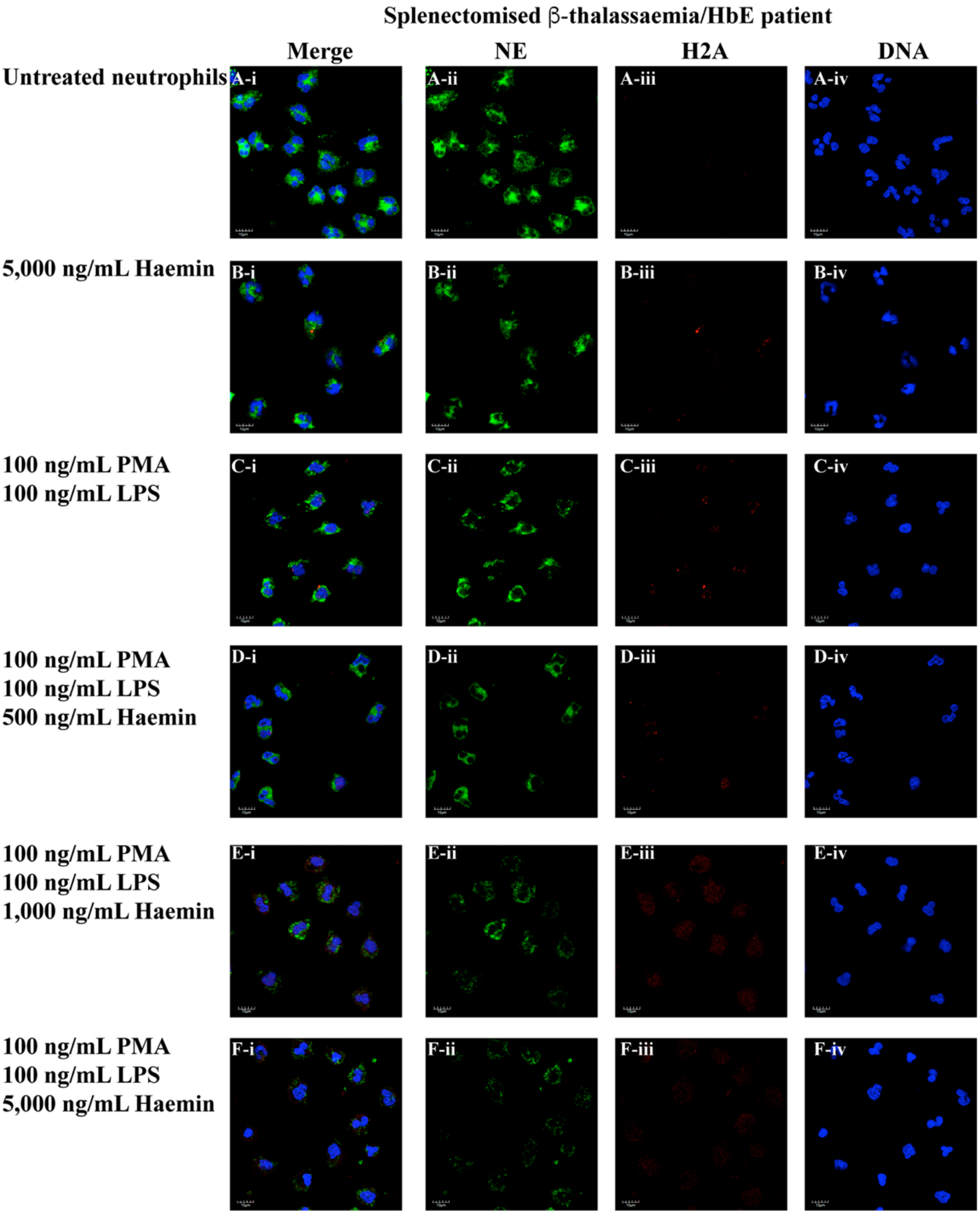

Supplementary Figure 4. NET formation in splenectomised β-thalassaemia/HbE patients. Isolated neutrophils from peripheral blood samples were incubated with absence or present of combined haemin and PMA/LPS for 90 min at 37°C, 5% CO_2. (Ai-iv) Untreated neutrophils, (Bi-iv) haemin-treated neutrophils and (C-F) combined PMA/LPS and different doses of haemin treated neutrophils were stained with fluorochrome conjugated antibodies specific to neutrophil elastase (NE, green), histone H2A (red) and nuclear DNA (blue) to analyze fluorescent intensity and morphology. i; merge, ii; anti-neutrophil elastase, iii; anti-histone H2A, iv; 4′, 6-diamidino-2-phenylindole, dihydrochloride (DAPI), LPS; lipopolysaccharides and PMA; phorbol 12-myristate 13-acetate.
